# What can be done to improve polycystic ovary syndrome (PCOS) healthcare? Insights from semi-structured interviews with women in Canada

**DOI:** 10.1186/s12905-022-01734-w

**Published:** 2022-05-10

**Authors:** Miya Ismayilova, Sanni Yaya

**Affiliations:** 1 Interdisciplinary School of Health Sciences, University of Ottawa, Ottawa, Canada; 2 School of International Development and Global Studies, Faculty of Social Sciences, University of Ottawa, 120 University Private, Ottawa, ON K1N 6N5 Canada; 3 The George Institute for Global Health, Imperial College London, London, UK

**Keywords:** PCOS, Polycystic ovary syndrome, Interviews, Lived experiences, Improvement, Reform, Health care, Health services

## Abstract

**Background:**

Polycystic ovary syndrome (PCOS) is a common and perplexing condition affecting metabolic, reproductive, cardiovascular, and psychological health in women. Previous studies point to widespread dissatisfaction and frustration in women with the information and care they receive. Studies have found delays with the diagnosis of PCOS and gaps in knowledge in physicians regarding the diagnosis and management of PCOS. Little has been heard from women on what they think can be improved with PCOS care, especially in Canada. This qualitative study explores women’s experiences navigating the healthcare system and their insights on what could be improved based on their lived experiences.

**Methods:**

Twenty-five participants were interviewed remotely over the phone by the first author between October and December 2018.Interviews were semi-structured and in-depth. Data were analyzed using thematic analysis and interpretive description methodology.

**Results:**

Twenty-five in-depth interviews conducted with participants across Canada (ages 18–63) revealed three overall areas in need of improvement. First, women emphasized a need for greater knowledge and awareness of PCOS in primary care physicians (PCPs) as well as the need for the medical community to prioritize women’s health. Second, participants advocated for greater PCOS awareness and de-stigmatization in the general community and in women and girls, and any individuals with female reproductive systems. Third, participants brought up several needed resources, such as the need for more PCOS research to be funded and undertaken, more PCOS specialists and experts to be available, credible doctor-provided information (e.g., pamphlets, websites), and age-specific support groups and mental health supports to be available. Participants were generally unaware of existing PCOS organizations and brought up the need for established PCOS organizations to aid in the training and retraining of doctors and local awareness-building in communities.

**Conclusions:**

Participants believed that PCPs in Canada needed to be well-versed on how to diagnose and manage PCOS to prevent delays in diagnosis and provide easier access to care. Further, greater awareness and de-stigmatization in the general community are needed so women can identify symptoms early and have access to support from those around them. Overall, PCOS may be an overlooked and under-prioritized condition, both in the Canadian healthcare system and general community.

**Supplementary Information:**

The online version contains supplementary material available at 10.1186/s12905-022-01734-w.

## Background

Polycystic ovary syndrome (PCOS) is a condition affecting 8–13% of women of reproductive age [1]. As a complex and multifaceted condition, PCOS impacts women’s health and well-being in a multitude of ways. Women with PCOS often struggle with menstrual irregularity [2], infertility [3], body image issues [4], eating disorders [5], hirsutism [6], acne [7], and anxiety and depression [8]. Insulin resistance, central obesity, and dyslipidemia are also prevalent and can place women at a higher risk for developing type II diabetes and cardiovascular disease [9, 10]. PCOS can also cause an increased risk of maternal, fetal, and neonatal complications, such as preeclampsia, gestational diabetes mellitus, and spontaneous preterm birth [11].

PCOS is diagnosed most often with the Rotterdam criteria when 2 out of 3 of the following features are present with the exclusion of other conditions: polycystic ovaries on ultrasound, biochemical/clinical hyperandrogenism, and oligo-amenorrhea [12]. First-line treatments for PCOS include lifestyle management and oral contraceptive pills [13, 14]. Lifestyle management, involving weight loss or healthy weight maintenance, remains the most effective first-line therapeutic intervention [15, 16].

There appear to be widespread gaps in knowledge and inconsistent approaches among residents and physicians on the diagnosis and management of PCOS [17–21]. A recent study with physicians revealed some difficulty and confusion with PCOS care, as the spectrum of PCOS presentation can make it hard to determine appropriate diagnosis and management [22].

Surveys and interviews with women have reported similar salient findings: women are dissatisfied with the information provided to them by physicians, face delays in diagnoses, and are frustrated with the levels of knowledge and effort their physicians have in addressing their concerns [23–28]. Some women lose overall trust with their physicians [25, 29]. Mental health impacts of PCOS are often unaddressed in doctor’s visits [28, 30, 31] and women describe being discounted or brushed off by physicians [25, 28, 29, 32, 33].

With such high rates of dissatisfaction and frustration reported by women with PCOS, along with gaps in knowledge and confusion in physicians, it becomes important to gather insights from this population on what can be improved in PCOS healthcare provision = . Little is known about women’s lived experiences navigating care for PCOS in Canada. This study explores women’s experiences accessing care for PCOS and describes their perspectives on how to improve healthcare delivery for PCOS based on their lived experiences. This study describes findings from in-depth interviews with women in Canada to improve the care they receive for PCOS and ultimately, their quality of life.

## Methods

### Study design

In-depth, semi-structured phone interviews with participants explored their experiences getting diagnosed with and managing PCOS, as well as their thoughts on how to improve PCOS healthcare based on their experiences. Interview participants were sourced from a larger, multi-methods study involving an online questionnaire about PCOS diagnosis experiences hosted on SurveyMonkey. Respondents who indicated interest in being interviewed at the end of their questionnaires were subsequently interviewed remotely, over the phone, on a first come first serve basis. Interviews were in-depth and lasted around 1 h (see Additional file 1 for interview guide). Only the themes related to participants’ views on how to improve PCOS healthcare are explored in this paper.

### Research setting

Survey respondents and interviewees participated from across Canada. Canada is a country with 38,005,238 people as of July 2020, with the 4 most populous provinces being Ontario, Quebec, British Columbia, and Alberta [34]. The three most populous provinces after Ontario (14,734,01) are Quebec (8,574,571), British Columbia (5,147,712), and Alberta (4,421,876) [34]. Interviews were conducted by the first author in a private residential office in Ottawa, Canada with no one else present.

### Participants and recruitment

A purposive convenience sample was gathered online with participants who met the inclusion criteria: age 18 years or older, reporting a medical diagnosis of PCOS, having lived in Canada since their diagnosis, and able to speak and understand English. No upper age limit was established to promote participation from older patients living with PCOS, in the peri- and post-menopausal stages of life. Participants were recruited through posts on PCOS groups on Facebook, Reddit, and online PCOS forums. Social media was used as a recruitment strategy to reach a wider sample from across all Canadian provinces. The survey was advertised entirely online by the first author posting a short paragraph about the purpose of the study along with a recruitment poster and a link to the survey on SurveyMonkey.

The PCOS Awareness Association also helped with recruitment by doing a one-time re-post on their Facebook page with the study’s recruitment poster and the survey link. Participants who filled out questionnaires were invited to submit their contact information for follow-up interviews. Participants were interviewed in the order in which their affirmative responses and consent forms were received. Recruitment continued until preliminary analysis during data collection suggested thematic consistency across age groups and no more peri- and post-menopausal women were available to interview. Recruitment took place between April and December of 2018.

### Data collection

Twenty-five interviews were conducted remotely over the phone by the MI between October and December 2018, averaging an hour in length and conducted in one uninterrupted meeting. All interviews were audio-recorded (with participant consent) and transcribed verbatim by the first author. One interview was held over Skype™ due to participant preference. Interviews were capped at 25 once data saturation was reached (i.e., no new codes were emerging) and no more participants in the 40 + age group were available for interview. Participants were encouraged to share their experiences through a semi-structured interview process. The semi-structured interview guide was developed by MI based on themes and gaps identified in previous literature, and included questions such as, ‘What do you think could be done to improve PCOS healthcare?’’ and ‘Are there any resources you wish were there for you?’ (See Additional file 1 for interview guide). Pseudonyms are used here and in all written documents.

### Data analysis

MI coded all qualitative interview data, including field notes made during and after interviews, and managed all data in NVivo 12 (QSR International Pty Ltd. Version 12, 2018). In accordance with Thorne et al.’s (2004) [35] interpretive description methodology, an inductive analysis technique was used to analyze data. Themes were derived entirely from the data. Thorne’s (2004) [35] interpretive description approach is widely used in nursing research and does not generate new truths or theories but rather describes thematic patterns and commonalities while also accounting for individual variations and provides a product that clinicians can use as a backdrop for clinical decision-making. Braun and Clarke’s (2006) [36] six key stages in the thematic analysis of qualitative data were also followed in this study: (1) Familiarize, (2) Generate initial codes, (3) Search for themes, (4) Review themes, (5) Define themes, and (6) Write up the data analysis. Codes, subsequent sub-categories, and over-arching themes were generated directly from topics raised in the data. To ensure inter-rate reliability and trustworthiness of the study, a second team member independently coded all interviews using free codes and rated data into various themes. Data analysis findings were audited by the coauthors and the categories were further refined.

### Trustworthiness

This study was reported based on the Consolidated criteria for reporting qualitative research (COREQ) (see Additional file 2) [37]. At the time of the study, MI was a MSc student conducting in-depth interviews for the first time after training in graduate classes and workshops. MI identifies as female, and participants were made aware of the reason for the author to be conducting this research, their personal interest in the research topic, and PCOS status, but otherwise no significant relationship existed or was established between the author and participants. To ensure reliability and validity, MI considered researcher bias, used the strategies of thick description, development of a coding system, checking and agreement on themes and analysis by members of the team, transparency when reporting research (as per COREQ), and demonstrating the author’s interpretive lens throughout the report [35, 37].

## Results

The interview sample of 25 participants included mostly White/Caucasian women born in Canada (see Table 1 for demographic characteristics). Most participants were between the ages of 25–30, resided in Ontario, and were employed full-time. Seven participants had children, and nine participants were looking to conceive at the time of the interview. All participant names below are pseudonyms. For information on participants’ diagnosis characteristics, see Table 2.

**Table 1:** Demographic characteristics of interview participants (*n* = 25)

Demographic characteristic	Number of women (%)
Age group	
18–24	5 (20)
25–30	10 (40)
31–36	4 (16)
37–40	2 (8)
41–50	2 (8)
51–66	1 (4)
Province	
Alberta	4 (16)
British Columbia	4 (16)
Ontario	13 (52)
Quebec	1 (4)
Ethnicity	
Black	1 (4)
East Asian	2 (8)
Middle Eastern	2 (8)
South Asian	2 (8)
White/Caucasian	18 (72)
Marital status	
Single	11 (44)
Common-law/live-in partner	5 (2 0)
Married	9 (36)
Education	
Bachelor’s degree	12 (48)
Master’s degree	3 (12)
Trade/technical/vocational training	2 (8)
Employment	
No paid work	3 (12)
Student	7 (28)
Employed full-time	12 (48)
Employed part-time	1 (4)
Parental status	
No children	18 (72)
Has children	7 (28)
Looking to conceive	9 (36)
Pregnant	2 (8)

Based on information participants were comfortable divulging, not all participants are captured

**Table 2:** Diagnostic characteristics of interview participants

Participant (pseudonym)	Age	Length of time since diagnosis	Diagnosing physician*	Number of physicians seen before attaining diagnosis	Length of time seeking diagnosis
Sally	30	1 month	Gynecologist	2	2 years
Melissa	34	1 year	RE	3	3 months
Mei	18	1 year	Pediatric gynecologist	3	2.5 years
Melanie	25	2 months	GP	2	3 months
Lily	18	1 year	Gynecologist	2	3 months
Lizzie	27	10 years	Pediatrician	2	4 years
Pam	28	6 months	Gynecologist	2	2 weeks
Josie	27	2 years	Gynecologist	2	10 years
Zara	24	2 years	Ob-Gyn	2	4 months
Abigail	31	2 years	Ob-Gyn	2	1 year
Brianna	21	5 years	Pediatric gynecologist	2	1 year
Fiona	31	5 years	Endocrinologist	2	4 years
Eileen	47	21 years	GP	1	6 months
Divya	22	2 months	Gynecologist	2	4 years
Mary	27	5 months	Endocrinologist	2	9 months
Bianca	36	5 years	GP	1	3 months
Jamila	26	1 year	GP	3	10 years
Holly	29	7 years	GP	1	1.25 years
Margaret	33	9 months	GP	4	10 years
Vanessa	63	43 years	GP	1	2 weeks
Rita	38	2 months	Fertility specialist	5	5 years
Patricia	29	8 years	GP	2	2 years
Lucy	47	14 years	Endocrinologist	6	10 years
Emma	29	5 years	GP	1	4.5 years
Josephine	32	3 years	Fertility specialist	2	3 months

^*^GP: General practitioner, RE: reproductive endocrinologist, Ob-Gyn: obstetrician gynecologist

**Fig. 1:**
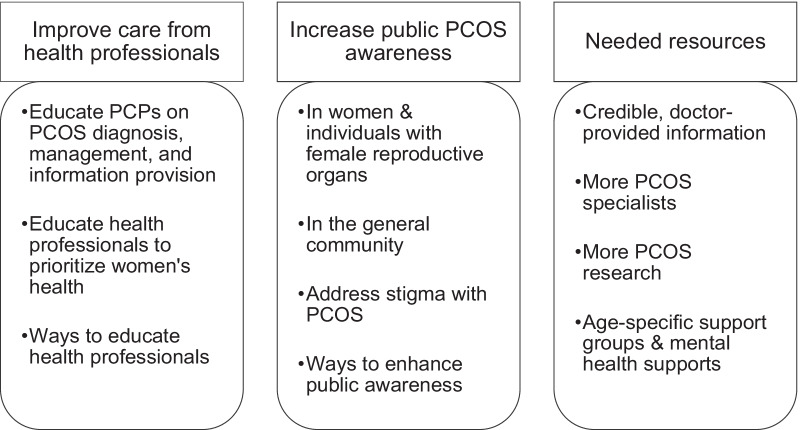
Themes and sub-themes on how to improve PCOS healthcare

Several themes emerged on how to improve PCOS healthcare. The main over-arching themes of areas of improvement include: (1) improvement of care provision from healthcare professionals, (2) promotion of public PCOS awareness, and (3) provision of several needed resources. Participant-identified strategies on how to bring about education reform for the public and medical community are also discussed. For a breakdown of the themes and sub-themes, please see Fig. 1.

### Improve care from health professionals

#### Educate PCPs on PCOS diagnosis, management, & information provision

One of the main topics participants highlighted was the need to improve PCOS healthcare by ensuring that primary care physicians (PCPs) can be more involved in care provision. Participants often began their diagnosis and management journeys with their PCPs, whose level of knowledge on what to do with their symptoms greatly influenced their experience. Many participants had long journeys to their diagnosis and described instances of PCPs brushing off concerns, not providing referrals, and not ordering tests to investigate their concerns.

Bianca, aged 36, was one of a few participants who was able to attain a diagnosis within 3 months and only saw her GP throughout her diagnosis and management. She had a positive experience accessing care and believed that PCPs should be well-versed on PCOS to ensure speedy diagnoses and ease of access to care:

I do not know how much education any physician generally has of PCOS. Obviously probably reproductive endocrinologists have a lot but as general physicians are those first contact probably diagnosing or starting the diagnostic process point of contact for people. I would think that that would be very valuable for those general physicians to be to be really well versed. So increased education for them as well.

Mary, aged 27, also discussed how PCOS is a prevalent condition in women and warrants knowledge at the PCP level:

So it's not this kind of rush to get like - it's not like you have to get a referral to get this diagnosis. Because really the frequency that it happens, it shouldn't be something where you need to wait for a specialist to get the first step of care. And that's kind of what it is right know. And if you can't get an endocrinologist for whatever reason. And I know in Canada we're fortunate to have covered healthcare. But in the States or somewhere where these things cost a lot of money then you might not ever get a diagnosis if you can’t afford to go to those specialists. So there's definitely something wrong when your general practitioner can't at least take the first steps to help you with your diagnosis.

Many participants’ PCPs were generally uninvolved with PCOS management. Brianna, aged 21, wanted more access to knowledgeable PCPs who could work alongside specialists in management of PCOS. She explains how important GPs are in healthcare delivery and the benefits of them working with specialists in the field to provide the best possible care:

It’s just also someone who actually knows your particular issue. They have all your methods and they have all the records of your issues. They see things that specialists don’t because they don't know your personal situation. So maybe a combination of doctors who know their situation and the doctors who are reading in this field.

Some participants wanted PCPs to know how important information provision is to them. Many participants (13/25 or 52%) were diagnosed without receiving enough information from their physicians on what PCOS is and how it may impact their lives. Patricia, aged 29, remarked that GPs needed to be encouraged to provide information to the patient at the time of diagnosis:

Because I guess they're usually the frontline people, make sure that they are aware of side effects and make it a point to tell them that you know “when you diagnose someone you should be giving these facts and these things as they go about their diagnosis.” I don’t know, maybe have a pamphlet or something that they can hand out so they can go over the effects and their treatment options and things like that.

It was important for participants to receive information from their physicians instead of having to search online to educate themselves. Participants expressed that although they wanted to trust their doctors, most lost trust due to the lack of information and/or involvement from their physicians. Mary, aged 27, expressed that she wanted to be able to trust and rely on her doctor, but when she was not finding the information she needed, it was hard for her to do so:

Because it's not that I wouldn't trust my doctor if they gave me that information but they don't. So it kind of instills a lack of confidence, you know? So it's not even “who do I trust the most” it’s like “who is the only one giving me information?” Because I would trust – like if my doctor handed me a pamphlet and was like “this is PCOS, if you have any questions ask me” then I would 100 percent trust that. But then it's like they're not doing that.

#### Educate health professionals to prioritize women’s health

Most participants felt that women’s health was not prioritized or taken seriously by the medical community, and PCOS being a women’s health issue, was also not taken seriously. Participants wanted the medical community to realize that they had been overlooking women’s health and thus, impacting healthcare delivery for PCOS and other conditions like it. As Mary, aged 27, put it, “*I feel like in general women's health has been neglected and this is just kind of like one of the issues that goes along with that*.”

Margaret, aged 33, explained how hard it can be to access the care you need for a women’s health condition. She explained:

I mean I don't think that that's just confined to PCOS. I think that that's something that you can probably say about women's health in general. I know I heard so many stories from so many of my other girlfriends and not strictly PCOS-related but sort of with chronic conditions that they dealt with, that it's like a fight really to find somebody. It seems like when you're a woman that it's that uphill battle of getting somebody to take you seriously. So I think that that's probably definitely a factor.

Abigail, aged 31, spoke about how often patients with PCOS push for a diagnosis and for their concerns to be taken seriously:

And you know probably not all deal with it in the same way because the symptoms are spread across the board for it. So I just think maybe more knowledge for all the doctors that it is, you know, an actual thing. Because I know some people go untreated for years and their doctors are like “no, you don’t have anything, nothing is wrong with you” and then all of a sudden, they have it. I’ve read a lot that people have to advocate that they have something and that their doctors have to keep testing them for stuff because some say “no you don't have anything, nothing is wrong with you, no.”

### Increase public awareness of PCOS

#### In women & individuals with female reproductive systems

Although the medical profession could use a lot more education about PCOS, participants also acknowledged that PCOS awareness in women is very low. Many participants identified a need for greater education and awareness on common women’s health conditions like PCOS. Drawing from their own experiences and what they wished was different in their journeys, participants reflected that greater awareness of PCOS might have allowed them to achieve a diagnosis sooner and start managing symptoms.

Lily, aged 18, reflected on how little discourse there is on PCOS and other women’s health conditions. She explained:

And not everyone knows about it. But I do feel like, in terms of health and sexual education, we can definitely start talking about these things. And hopefully people will be more educated and be likely to seek medical help if they actually do experience symptoms.

Josephine, aged 32, remarked how little knowledge her circle of friends, and herself, had of PCOS. Josephine reflected that maybe she might have been diagnosed with PCOS sooner had she been aware of it:

To speak more about it because I read that a lot of women have PCOS. It was one of the first reason for infertility for example. But when I discovered I had PCOS I talked about it with some friends around me but nobody ever heard about it. And I think if I've heard about it before maybe I would have discovered that I have PCOS a long time ago because of my weight, acne, and my periods. And I wouldn’t have waited so many years to have answers. And I think there was a lot of women who have PCOS and they don't know about it.

#### In the general community

Another benefit to building general community awareness of PCOS is the ability to make women with PCOS feel less isolated and alone. One of the most common descriptors participants used to explain having PCOS was “isolating/alone.” Most participants had little to no real-life friends who were aware of PCOS, despite it being a relatively common condition, which made participants feel more isolated dealing with their condition on their own.

Lily, aged 18, felt hesitant to open up about her diagnosis and once she did, she found that none of her friends understood what it was or were aware of it:

At first, I didn’t really talk about it or tell anybody other than my parents, but yeah now that like I’ve talked about it more with people my own age… A lot of people don’t really know what PCOS is, and there are a lot of misconceptions about it, I guess. And just about other menstrual problems that come along with it. There was just not a lot of conversation about it, I guess… I didn’t know anything about PCOS until I was diagnosed with it. It’s not something that’s taught in schools I guess

Pam encountered people around her to not be concerned about or aware of PCOS, she describes it as being a lonely experience:

I think it’s an education thing, people aren’t that concerned about it. It’s probably just because they don’t know about it. PCOS takes so many forms and it can be so severe or not so bad, and I feel fortunate that I’m not struggling with diabetes as a result of PCOS. It could be so much worse. It has been definitely a lonely experience.

Divya, aged 22, reflected a lot on societal lack of awareness of PCOS and the potential drawbacks it can have on women, making them feel more isolated and misunderstood. She explains:

I can understand people saying like “oh no one really even knows what it is, and they have no empathy in the public for it.” Because actually like you see online a lot… like there’s that one obese model or something, Whitney or someone from “My 600 pound life.” But she has PCOS. And everyone makes fun of her for her weight. They don't actually think that it's harder to lose weight on PCOS. So I see a lot of that, and that kind of sucks too where everyone's like “oh you people use PCOS to hide behind their weight issues” but it's like it's actually harder because of our insulin issues but no one really knows that.

Divya referred to PCOS being linked to various mental illnesses and eating disorders and thought that societal unawareness may contribute to greater stigma around those aspects of PCOS:

I know a lot of people with PCOS have problems with binge eating or eating disorders or mental health like depression symptoms. Either due to their symptoms or just like the societal like no help at all for what they're dealing with.

Awareness of PCOS and other women’s health conditions was identified as important in general physical and sexual education. If the general community were more aware, women would be more likely to spot concerning symptoms and access care promptly, parents would know to take their children in to the doctor, and people would be able to support one another with understanding.

### Address the stigma with PCOS

A lot of participants reflected on there being a stigma around PCOS (and potentially women’s health in general) when they noticed themselves feeling shameful about talking about their symptoms openly, or they noticed others around them not discussing PCOS openly. Many PCOS symptoms like acne, excess hair, weight gain may be perceived as undesirable and less feminine in women, which introduce stigma into having this condition. There does appear to be a real stigma with women’s health that needs to be lessened as awareness of the condition is established.

A lot of participants found themselves hesitant to openly share their diagnosis and symptoms—and were often not sure why. Lily, aged 18, described her thoughts:

I guess there’s a stigma around it - you don’t really want to tell people and I feel like just in general there’s a stigma around periods. But yeah, I still don’t feel 100% comfortable telling my friends about it. And not everyone knows about it.

Patricia, aged 29, detailed how she experienced unwanted facial hair and has experienced people around her making fun of others with facial hair, leading her to keep her symptoms to herself:

I am someone who is terrified of waxing so I just shave it daily which is sort of embarrassing but you deal with that. Some are cool with that. And then some other people who don't shave their faces or take care of the hair, which I'm totally fine with them doing whatever they like. But I do have some good friends that I haven't really shared that portion of my symptoms with them. And to hear them kind of make fun of other people with it, it’s always like “well it's not always their fault.”

PCOS symptoms like hirsutism and weight gain are typically ridiculed and deemed undesirable in women by society’s current standards, which contributed to women keeping their symptoms to themselves, as Patricia did. Pam, aged 28, consciously chose to speak out about her condition, in hopes to lessen the stigma and increase awareness:

It’s been the most surprising part of it. The statistics are like, I mean it varies, but say 1 in 10 women have PCOS. And I think because it’s related to the sex organs, there’s a bit of shame about it or secrecy… It’s a private thing, people don’t talk about it. But I’ve been really vocal about it because I think the odds are 1 in 10 women have it, like some of my friends might have it, they just never talked about it because they never felt comfortable. And it can be very isolating dealing with PCOS… But a lot of people have struggles like PCOS but feel shy talking about them for whatever reason.

### Ways to enhance physician knowledge and public awareness about PCOS

Participants identified numerous ways to enhance physician and public awareness about PCOS. Table 3 presents the key take-aways:

**Table 3:** Themes on how to raise physician and public awareness of PCOS

Theme	Sub-themes and quotations			Remarks
Ways to increase physician knowledge about PCOS	**Conferences** *Divya, aged 22: “Maybe like a workshop or conference that talks about the prevalence in women and what the symptoms are and how they could help if there is any research.”*	**Seminars/webinars** *Fiona, aged 31: “I wish that like there was a seminar or something. And also I wish there was like a doctor seminar so that my actual doctor could take a class on it or go to an information session or read some case studies or I don't know. Because I know that people learn from other doctors. So I wish my personal doctor could go to that. Or have the opportunity to see stuff.”*	**PCOS organizations** *Lucy, aged 47: “Well if there was some sort of organization that provided that information on a regular basis, where professionals who had to work with women who have PCOS, and there are a lot of them out there, the GPs and specialists who are required to take this ongoing training and to be updated so that their information was valid and up to date. I think that would be a positive thing.”*	Despite the need for them, there are not many well-established PCOS organizations that host a lot of educational and awareness-building events and activities in Canada. Very few participants recalled visiting the websites of PCOS organizations and finding relevant information
Ways to enhance public PCOS awareness	**Health and sexual education class** *Lily, aged 18: “I would definitely like to see things like PCOS, endometriosis, things that are not just what a normal regular period should be like but maybe like how abnormalities are. Like what to do if you’re experiencing symptoms like the irregular menstrual cycle I guess… So being integrated in sex ed or in elementary schools especially. During that time we’re first experiencing health class.”*	**PCOS organizations** *Divya, aged 22: “Like I feel there’s a lot of awareness on breast cancer, and fundraisers and everything around campuses, packages of food, and pens, and whatever you like to see. There’s no types of things for PCOS. You always hear about those other diseases but I’ve never really seen a PCOS-centered, like “this is a run for women with PCOS.” Even though I feel like it’s 1 in 10, like many women have it now. So it's not uncommon, it's not like a niche to say “let's do a PCOS-targeted type of fundraiser.””’*	**Local, community-level advocacy** *Josie, aged 27: “I think the idea of having a more local group advocating for PCOS education or something like that. I know there’s fairs and festivals and different events that we have locally that could include a little booth for PCOS. I think just getting into the community, having people who can share what they know, share resources that they’ve been given or found, yeah. I think starting at the local level is helpful.”*	Raising PCOS awareness in the community was a very prominent theme raised by all participants. Raising awareness was important to help women take charge of their own health, to break the stigma surrounding women’s health, and to have less women feel isolated when living with PCOS

### Participant-identified need resources

A few needed resources came up for participants, such as the need for doctor-provided information and support groups. Table 4 summarizes the key themes.

**Table 4:** Participant-identified needed resources

Theme	Sub-themes and quotations		Remarks
Credible, doctor-provided information about PCOS	**Pamphlets, websites** Holly, aged 29: *“But I mean if the doctors could just have a pamphlet or direct people to these sorts of things, that would be great… Even like a pamphlet, it's just understanding that like irregular menstruation is not the only thing that happens. You can have weight gain, you can have insulin resistance, you'll have cystic acne, and that sort of thing. Not just by doing all my own research I'm finding out all these different things.”*	**Multilingual formats** Lily, aged 18: *“With my parents, I guess there’s a slight language barrier as they speak Chinese and I speak English primarily. My Chinese isn’t amazing… I found it really difficult to have the conversation with them because of the language barrier. I would definitely have appreciated multi-language resources. Really, the only resources I found were in English. But I’m not sure if that’s because I only speak in English, I can only read in English. It would be extremely beneficial if I had those resources for languages.”*	Take-home reading material, in multilingual formats, is needed so that even if the doctor does not have much time for a sit-down, the patients can learn on their own time from a reliable source
More PCOS specialists	**PCOS clinics** Emma, aged 29: *“There are no clinics they send you to when you're diagnosed with PCOS where they're like this is a clinic where they have actual experts in this topic that you can meet with… It would be very nice if there was like "oh there's this PCOS clinic and you can be referred to this and then they will do things like look at your hormone levels and they will know to ask those questions." That would be very very helpful because it would give you the ability to manage your body and your disorder by letting you refer to experts without having to start from square one… So I think that it would be very nice if there were more specialists in reproductive health issues that you could see well before you were actively trying to get pregnant.”*		A lot of participants had positive experiences with fertility specialists, who they found to be well-versed on PCOS, perhaps due to seeing many patients with PCOS seeking fertility treatment. A need arose for greater access to PCOS specialists, in PCOS-specific clinics and otherwise
More PCOS research	**Treatments** Jamila, aged 26: *“But I really wish that someone would actually be able to find some cure for it. And to do more funding to find more research on it. On the Internet, it's the same thing that I keep finding being repeated but hardly much new things are there. So that's the frustrating part, not being able to find an answer.”*	**Menopause** Lucy, aged 47: *“I have tried to see if there's any research out there on it and there is virtually nothing on PCOS and menopause or perimenopause… What we forget is this isn’t a condition that goes away. It stays with you. And the little research I have found is that “oh yeah symptoms get worse when you go into menopause” and suddenly you're dealing with even more severe—you know how they say diabetes and heart disease are a problem when you have PCOS, well it even ups that much more when you are in menopause.”*	Participants wanted more research to come out about PCOS treatment options to help them understand and manage their condition better. Especially with regards to treatment options for women with PCOS in peri- and post-menopause
Age-specific support groups & mental health supports	**Age-specific support groups** Vanessa, aged 63: *“I think it would need to be age appropriate first. Like I wouldn't want to hang out with a bunch of 20-year olds. I think their needs are different. Their focus is different. But yeah I would like something like that.”*	**Counsellors with an understanding of PCOS** Lucy, aged 47: *“I would have appreciated a support group and even counselors who had an understanding at least of PCOS to talk to. I think that probably would have helped the most.”*	Local support groups were a service that many women wanted to be provided by either organizations or health providers. Specifically, support groups for women in a similar life stage and/or with similar treatment goals. Some participants also wished for counsellors or doctors to be present at the support groups to provide professional advice and learn from patient experiences

## Discussion

Many participants argued that for a condition as common as PCOS, knowledge at the PCP level was needed to address their health concerns more efficiently. Participants believed that a knowledgeable and well-versed PCP could speed up diagnoses for patients by avoiding the need for referrals to specialists and help them garner easier access to whichever resources they needed throughout their management journeys. Participants especially emphasized that PCPs need to be more aware of the spectrum of PCOS presentation – the spectrum of presentation is a challenge for many women and physicians with regards to management [22].

A recent qualitative study has recommended for PCPs to be more involved with PCOS management [31]. Little is known about levels of knowledge of PCOS in primary care. Most studies pointing to significant gaps in knowledge in physicians have examined obstetrician-gynecology and endocrinology residents and physicians, finding many not knowing which PCOS diagnostic criteria they used or being unable to correctly identify diagnostic criteria [17, 19, 21]. A 2018 study found that women had greater levels of distrust in their PCP’s opinion and felt that they spent less effort treating their PCOS concerns [25], and another study found that women received little information about long-term PCOS implications from their GPs [30].

A lot of participants felt brushed off and not taken seriously by their doctors when bringing up symptoms, which happened most often in visits with their GPs but also with specialists. Women recalled hearing stories about others with PCOS also not being taken seriously by doctors until symptoms escalated or years had passed. Participants identified a need for educational reform for physicians to include an emphasis on taking women’s health seriously to account for a potential under-prioritization of women’s health. Previous studies have found that women with PCOS feel discounted by their physicians [25, 28, 29, 32, 33]. One study found incongruences with what women with PCOS reported to be their biggest symptoms of concern versus what physicians perceived to be concerning for patients (e.g., pain was the most common concern for patients which clinicians did not consider to be important) [38]. Health professionals may need to take greater care to understand and investigate patient concerns.

Participant-identified ways to educate and raise awareness in physicians included the involvement of established PCOS organizations, or professional societies, who could provide up-to-date training or informational packets for physicians. Webinars, expert panels, and conferences were identified as ways to engage physicians – some participants preferred them being open to the public so that they could also attend and pick up useful information. A few articles and guidelines have been published in recent years guiding management of PCOS in primary care [39, 40] and international guidelines recommend healthcare delivery for PCOS in primary care as it is well-placed to diagnose and coordinate interdisciplinary care for patients [14]. A recent study on pediatric PCPs caring for PCOS found variability in beliefs around ability to diagnose and manage PCOS [41], suggesting that wider dissemination of guidelines may be needed.

Prior studies have also reported a need for evidence-based information in women with PCOS [24–26, 42–44]. A recent study has found that PCOS-related Google searches have steadily increased over the years [44]. Pamphlets were a resource that participants thought that doctors could quickly provide to participants, saving time for the doctor and providing participants with take-home material that they could digest after any shock from the diagnosis wear off. One participant of Chinese background hoped that as informational sources are developed, multilingual versions become available so that she and other multicultural patients can share what they learn with family members. Ethnicity and education level need to be accounted for when designing informational resources for women [45].

Numerous participants expressed the need for more physicians to specialize in or take interest in PCOS, as well as for more PCOS-centric clinics to open. Very few participants had visited or heard of a nearby PCOS clinic or PCOS expert to contact. There are few established multidisciplinary PCOS treatment clinics globally; however, studies are coming out to support the merit of a multi-practice approach and the need for more of these clinics [46]. In Canada, there are a few PCOS clinics including the Polycystic Ovarian Syndrome Clinic in the Women’s College Hospital and the Endocrine and PCOS Clinic, both located in Toronto. PCOS experts may be far and few in between; telemedicine and other types of digital consultations with specialists may be possible options to provide women with access to more experts [29].

Some participants were also frustrated at there being no cure for PCOS and the number of unanswerable questions around PCOS. A recent qualitative study also reported frustration in women around the lack of treatment options offered and unanswered questions about management [22]. Participants could not find an accessible source that was on the “cutting edge” of PCOS research. A general need came up for more PCOS research to explore therapies and to be open and accessible for women to read and/or participate in.A recent study estimates that PCOS receives less than 0.01% of national funding in the U.S. and may be underfunded considering its prevalence, economic burden, metabolic morbidity, and impact on quality of life [47].

Participants often described relying on online communities and/or friends and family for support when dealing with challenges relating to PCOS. Many noted that a local support group would be a beneficial resource to have. Identified benefits to support groups included finding others with PCOS to connect with, learning from and being motivated by other women’s treatment strategies, and sharing a safe space with others with similar lived experiences. Older participants communicated that health concerns and lived experiences with PCOS varied with age and that they would benefit most from attending age-specific support groups with others in similar situations.

Previous qualitative studies have also showed that women have a need for social support and connection with other women with PCOS [27, 48–50] and support groups often enable women to do just that, whether that be online or locally [27, 29, 33, 51, 52]. A study evaluating the effectiveness of a nurse-led support group left participants feeling more empowered in self-management and benefitting from informational exchanges [53]. The 2018 International Evidence-Based Guideline for PCOS acknowledges that PCOS support groups could be a key resource for addressing current gaps in information provision [14]. Few studies have examined the needs of older participants with PCOS and age-specific support groups. More research is needed to better understand the specific information and socio-emotional needs of women of different age groups with PCOS.

Several participants noted that counsellors with an understanding of mental health and PCOS may be a beneficial resource for them, particularly at support groups to better lend a safe space for women to access support for their symptoms. A previous study on group counselling for women with PCOS found that participants grew in motivation from the supportive environment and the follow-up exercise sessions, which led to significant post-intervention reductions in weight and BMI [54]. Partnerships between support groups, health professionals, and academics have been proposed to be used to strengthen the information provided in support groups; however, more research is needed to assess which benefits such partnerships can provide for women [55].

A distinct theme came up in participants across all age groups on the need for greater PCOS awareness – in the general community and in women, girls, and individuals with female reproductive systems. Greater awareness of PCOS could allow women and girls to better understand their symptoms and know when and how to check up on them. Recent studies have found lower levels of awareness of PCOS in young women and the public, urging for a need for greater awareness programs to be implemented [56–60].

Participants also stressed that the general community needed to be made more aware of conditions like PCOS so that they can be of support; the most common descriptors used by participants to describe their lives with PCOS were “isolating” and “alone.” Several other studies have also found women feeling isolated and alone having PCOS [29, 31, 33, 53, 61].

Raising awareness in the general community could facilitate a reduction in the current stigma around PCOS [62]. Participants rarely found themselves being open about their symptoms, described feeling a sense of stigma within themselves and in those around them, especially around the “unfeminine” or “undesirable” aspects of PCOS, similarly to participants in other studies [26, 29, 62, 63]. Some participants recalled seeing ill-informed and/or stigmatizing comments made about PCOS and similar conditions online. A study on media depiction of PCOS in magazines found that PCOS symptoms were largely presented as a hindrance to women’s roles as wives and mothers, and primarily portray white women with PCOS [64]. More research into how PCOS is portrayed in popular media is needed, as well as efforts to ensure that an accurate, unstigmatized, and diverse view is presented.

Women’s health overall was perceived by participants to be shrouded in unnecessary mystery. The ways in which participants thought to bring about greater awareness of the condition included revisions to sexual education curriculums to include a brief overview of PCOS and other common women’s health conditions, and for PCOS organizations to organize local events and fundraisers to build up awareness. Almost none of the participants recalled hearing much about local Canadian or American PCOS organizations – an opportunity exists for PCOS organizations to scale in their reach and advocacy efforts, with appropriate funding.

## Limitations, strengths, and positionality

Limitations of the study include the sample size and selections bias due to all participants being recruited from websites and online groups, eliminating the potential to reach women with PCOS who are not as active online. Most interview participants identified as Caucasian; few participants from diverse ethnic backgrounds were captured. Little understanding can be drawn about how PCOS healthcare can be improved for individuals with different social identities who may have unique experiences and need culturally- and/or gender-sensitive health care services [65].

Strengths of this study include the involvement of peri- and post-menopausal women whose experiences and beliefs may not be transferable to the experiences of women of reproductive age. The use of remote phone and Skype interviews allowed participants to feel comfortable and save financial and time costs by interviewing from their preferred locations. Overall, this study addresses an important gap in the literature on women’s experiences with PCOS healthcare in Canada and their perspectives on what can be overall improved based on their lived experiences.

With regards to the interviewer’s positionality, MI had shared with participants her prior knowledge of PCOS due to having friends with PCOS, but that she herself did not have a PCOS diagnosis. Participants may have felt more reserved with MI than if they had been interviewed by someone with PCOS; however, disclosing that MI was personally connected to friends with PCOS, may have helped participants feel more understood. As MI was a student and novice researcher, who was not involved in the healthcare profession, participants may have felt more at ease being open about their experiences navigating the healthcare system without the presence of a potential power dynamic.

## Conclusion

Adequate knowledge and awareness of PCOS diagnosis, management, and biopsychosocial aspects in PCPs is necessary for timely diagnosis and meaningful treatment plans. Our findings support prior recommendations to provide women with evidence-based information on PCOS features and management, in pamphlet form or as a list of credible web sites, and contact details of PCOS support groups as necessary [24, 66, 67]. Referral to support groups may be important for some patients to reduce feelings of isolation and connect women to a community where experiences and information can be shared. Women also identified a need for greater awareness and de-stigmatization of PCOS for the general community, and greater prioritization of women’s health in the medical community.

### Policy recommendations and future research

Participant experiences revealed that some things are amiss with PCOS healthcare delivery and clinical shifts are necessary to provide appropriate care for this population. To address gaps in knowledge and awareness, professional societies should provide relevant educational materials that can be easily accessible to patients and physicians. International evidence-based guidelines and reviews [14, 39, 40, 68, 69] have been established for the diagnosis and management of PCOS which can be consulted by clinicians in practice. The guidelines inform of the necessary information to provide and of the importance of mental health screening, among other screening and testing to be done, accounting for the needs of different phenotypes and age groups of women. Evidence-based translation and education resources for physicians and women with PCOS, along with the first, evidence-based app for women with PCOS “AskPCOS,” can be found at https://www.monash.edu/medicine/sphpm/mchri/pcos/resources. Greater dissemination of these evidence-based guidelines may be necessary to promote best practices.

Efforts are needed to raise awareness for PCOS in the general and medical communities in Canada. Several advocacy and policy efforts have been successfully undertaken across the world. The PCOS Challenge, an American advocacy group, has designated the month of September as the “Polycystic Ovary Syndrome Awareness Month” [70]. Greater advances have been made in Australia, where The Federal National Women’s Health Strategy 2020–2030 aims to raise PCOS awareness in the general and medical communities, and PCOS has been integrated into the 2017–2020 key priorities for women’s sexual and reproductive health [70].

Studies examining knowledge levels and practice patterns in Canadian PCPs are needed to address the gap in the literature. Future mixed-methods research could elicit additional insight on areas of improvement. Future research should explore the role of age-specific support groups in the management of PCOS and the benefits of health professional-led support groups. A list of current international English-speaking PCOS online support groups is made available by Avery et al. 2020 [55]. More research is needed to understand the lived experiences and needed healthcare services for individuals of various social identities and backgrounds; there is a lack of research in culturally- and gender-sensitive standards of care for PCOS [65]. Studies analyzing media content on PCOS can help researchers, patients, and stakeholders address stigma and engage with the media to increase public awareness of PCOS [65]. Studies on Canadian youth and public are needed to gauge awareness levels across socioeconomic strata and urban/rural regions. Finally, more research is needed to examine whether PCOS-related research is underfunded in Canada; policy changes may be warranted to address inadequate funding.

## Supplementary Information

**Additional file 1.** Interview Guide

**Additional file 2.** COREQ checklist

## Data Availability

The interview guide containing questions about the diagnosis is made publicly available, other data are available from the corresponding author on reasonable request.

## Abbreviations

COREQ: Consolidated criteria for reporting qualitative research
GP: General practitioner
Ob-Gyn: Obstetrician-gynecologist
PCOS: Polycystic ovary syndrome
PCP: Primary care physician
RE: Reproductive endocrinologist
