# Supplementary material for: What can be done to improve polycystic ovary syndrome (PCOS) healthcare? Insights from semi-structured interviews with women in Canada
Source: BMC Womens Health. 2022 May 10;22:157. doi: 10.1186/s12905-022-01734-w (PMC9092874; doi:10.1186/s12905-022-01734-w)
Supplement: Supplementary file 1 — Additional file 1. Interview Guide [file 12905_2022_1734_MOESM1_ESM.pdf]

Interview guide

Ways PCOS healthcare can be improved:

1. Could you tell me about any healthcare resources you personally use which have helped you on your journey?
2. Where did you hear about the resources you are using now?
3. Who or what do you trust most when looking for new information on PCOS healthcare?
4. Are there any resources you wish were there for you?
5. Could you tell me about any resources you might be aware of but have chosen not to use?
6. What do you think could be done to improve PCOS healthcare?
7. Is there anything else you would like to add?
