# Supplementary material for: What can be done to improve polycystic ovary syndrome (PCOS) healthcare? Insights from semi-structured interviews with women in Canada
Source: BMC Womens Health. 2022 May 10;22:157. doi: 10.1186/s12905-022-01734-w (PMC9092874; doi:10.1186/s12905-022-01734-w)
Supplement: Supplementary file 2 — Additional file 2. COREQ checklist [file 12905_2022_1734_MOESM2_ESM.pdf]

Consolidated criteria for reporting qualitative studies (COREQ): 32-item checklist:

**No. Item**	**Guide questions/description**	**Reported in Section**
**Domain 1: Research team and reflexivity**		
Personal Characteristics		
1. Interviewer/facilitator	**Which author/s conducted the interview or focus group?**<br><br>Miya Ismayilova conducted all interviews.	Methods, page 5
2. Credentials	**What were the researcher's credentials? E.g. PhD, MD**<br><br>The authors' credentials are as follows:<br>- Miya Ismayilova, HBSc<br>-Sanni Yaya, PhD	N/A
3. Occupation	**What was their occupation at the time of the study?**<br><br>MI : Master's student<br>SY: Professor	Methods, page 6
4. Gender	**Was the researcher male or female?**<br><br>MI: Female<br>SY: Male	Methods, page 6
5. Experience and training	**What experience or training did the researcher have?**<br><br>MI: quantitative and qualitative research training.<br>SY: quantitative and qualitative training and extensive experience in global maternal and child health, including sexual and reproductive health care.	Methods, page 6
Relationship with participants		
6. Relationship established	**Was a relationship established prior to study commencement?**	Methods, page 6

	No prior relationships was established	
7. Participant knowledge of the interviewer	**What did the participants know about the researcher? e.g. personal goals, reasons for doing the research.**<br><br>Participants knew about the reason MI was conducting this research, their person interest in PCOS, and PCOS status	Methods, page 6; Discussion, page 39
8. Interviewer characteristics	**What characteristics were reported about the interviewer/facilitator? e.g. Bias, assumptions, reasons and interests in the research topic**<br><br>MI's reasons and interest in the research topic was reported	Methods, page 6; Discussion, page 39
**Domain 2: study design**		
Theoretical framework		
9. Methodological orientation and Theory	**What methodological orientation was stated to underpin the study? e.g. grounded theory, discourse analysis, ethnography, phenomenology, content analysis**<br><br>This study uses Thorne et al.'s (2004) interpretive description methodology, which is widely used in nursing research and does not generate new truths or theories but rather describes thematic patterns and commonalities while also accounting for individual variations and provides a product that clinicians can use as a backdrop for clinical decision-making.	Methods, page 6
Participant selection		
10. Sampling	**How were participants selected? e.g. purposive, convenience, consecutive, snowball**<br><br>Participants were selected in a purposive convenience sample online in various PCOS groups and forums, along with the help of the PCOS Awareness Association who posted the recruitment poster on their Facebook page.	Methods, page 5
11. Method of approach	**How were participants approached? e.g.**	Methods, page 5

	**face-to-face, telephone, mail, email**<br><br>Participants were reached out to via email, and later by telephone when interviewed.	
12. Sample size	**How many participants were in the study?**<br><br>A total of 25 participants.	Methods, page 5
13. Non-participation	**How many people refused to participate or dropped out? Reasons**?<br><br>None	N/A
Setting		
14. Setting of data collection	**Where was the data collected? e.g. home, clinic, workplace**<br><br>Data collection took place at home where the interviewer was when interviewing.	Methods, page 4
15. Presence of non-participants	**Was anyone else present besides the participants and researchers?**<br><br>No one was present except for participants and MI who was interviewing.	Methods, page 4.
16. Description of sample	**What are the important characteristics of the sample? e.g. demographic data**<br><br>Participants ranged in age from 18 to 63 years old, with mostly Caucasian backgrounds. The length of time since the diagnosis of PCOS varied between 1 month to 33 years at the time of enrollment in the study. Most participants resided in Ontario, with a few from Alberta (n=4), British Columbia (n=4), and one from Quebec. Most participants were employed full-time, 7 were students, and 2 were stay-at-home moms. Seven participants had children, and nine participants were looking to conceive at the time of the interview.	Results, page 8.
Data collection		
17. Interview guide	**Were questions, prompts, guides provided by the authors? Was it pilot tested?**	Methods, page 4

	The interview guide was provided (see Supplemental File 1), it was not pilot tested.	
18. Repeat interviews	**Were repeat interviews carried out? If yes, how many?**<br><br>Repeat interviews were not carried out.	N/A
19. Audio/visual recording	**Did the research use audio or visual recording to collect the data?**<br><br>Interviews over the phone were recorded (only audio data was collected).	Methods, page 5
20. Field notes	**Were field notes made during and/or after the interview or focus group?**<br><br>Yes, reflective notes were made MI during and after all interviews.	Methods, page 6
21. Duration	**What was the duration of the interviews or focus group?**<br><br>Interviews lasted an average of 1 hour.	Methods, page 5
22. Data saturation	**Was data saturation discussed?**<br><br>Yes. Interviews were capped at 25 once data saturation was reached and no more participants in the peri- and post-menopausal stages were available.	Methods, page 5
23. Transcripts returned	**Were transcripts returned to participants for comment and/or correction?**<br><br>Transcripts were not returned to participants for comment/correction.	Discussion, page 39
**Domain 3: analysis and findings**		
Data analysis		
24. Number of data coders	**How many data coders coded the data?**<br><br>MI coded all data.	Methods, page 6
25. Description of the coding tree	**Did authors provide a description of the coding tree?**	Methods, page 6

	Yes. The transcript was read and coded based on identified similarities and patterns in the data. Codes, sub-categories, and over-arching code categories were generated directly from transcripts.	
26. Derivation of themes	**Were themes identified in advance or derived from the data?**<br><br>Themes were derived from the data.	Methods, page 6
27. Software	**What software, if applicable, was used to manage the data?**<br><br>NVivo 12	Methods, page 6
28. Participant checking	**Did participants provide feedback on the findings?**<br><br>No, it was not possible due to not asking permission to save their contact information and contact them for member-checking.	Discussion, page 39
Reporting		
29. Quotations presented	**Were participant quotations presented to illustrate the themes/findings? Was each quotation identified? e.g. participant number**<br><br>Yes, quotes were used and identified.	Results, pages 9-48
30. Data and findings consistent	**Was there consistency between the data presented and the findings?**<br><br>Yes.	Results, pages 9-31
31. Clarity of major themes	**Were major themes clearly presented in the findings?**<br><br>Yes, we organized the findings by major themes.	Results, pages 9-31
32. Clarity of minor themes	**Is there a description of diverse cases or discussion of minor themes?**<br><br>Yes, we discussed minor themes in the manuscript and situated them within the broader literature.	Results, pages 9-31; Discussion, pages 31-39
